# Perceptions of dental professionals and laypeople to altered dental esthetics in cases with congenitally missing maxillary lateral incisors

**DOI:** 10.1186/2196-1042-14-34

**Published:** 2013-10-01

**Authors:** Marco Rosa, Alessia Olimpo, Rosamaria Fastuca, Alberto Caprioglio

**Affiliations:** Department of Orthodontics, University of Insubria, Varese, Italy; Trento, Italy; Bergamo, Italy; Orthodontic Program, Department of Orthodontics, University of Insubria, Varese, Italy; Postgraduate School of Orthodontics, Department of Orthodontics, School of Dentistry, University of Insubria, Varese, Italy

**Keywords:** Congenitally missing maxillary lateral incisors, Smile perception, Smile attractiveness

## Abstract

**Background:**

The smile perception of patients is not strictly related to standardized protocols and technical implications which certainly affect clinicians' decisions. The absence of maxillary lateral incisors could affect smile esthetics either with treatment or not. The aim of the present study was to investigate if different perceptions on altered smiles due to missing maxillary lateral incisors, with or without treatment, exist among different groups of people (laypersons, adult orthodontic patients, general dentists, and orthodontists).

**Methods:**

An ideal smile model was selected and altered simulating different malocclusions and treatment options. Twelve simulations were submitted to four categories of respondents: laypeople, adult orthodontic patients, general dentists and orthodontists. They were asked to express smile perception for each simulation by ranking and rating simulations using a 0 to 100 visual analog scale. Analysis of variance was used to determine if there were statistically significant differences in values assigned among the four categories of respondents for each simulation.

**Results:**

Significant differences in smile perceptions were found between professionals (dentists and orthodontists) and laypeople. Presence of dental tipping and marked diastema in the arch were disharmonious aspects less tolerated in a smile by all categories of evaluators. Simulations associated with space closure orthodontic treatment were ranked as the most attractive smile and significantly ranked higher by dental professionals than patients and laypeople.

**Conclusions:**

Treatment, absence of diastema, and symmetry were the most accepted characteristics by all categories of respondents. Ideal orthodontic treatment options might be overestimated by clinicians when compared to laypeople's smile perception.

## Background

The prevalence of maxillary lateral incisor agenesis ranges between 1% and 3% [1], and maxillary lateral incisors account for approximately 20% of all missing teeth in Caucasian population [2]. Two treatment options could be considered in the case of congenitally missing maxillary lateral incisors. The space of the missing tooth could be opened, placing maxillary canine into its natural position and subsequently replacing the missing lateral incisor with prostheses [2–6]. On the contrary, orthodontists might close the space by repositioning the canine into the position of the missing lateral incisor and the first premolar into the position of the canine. The space closure alternative could be associated with tooth remodeling and restorations of the canine and often of the first premolar [7–11].

A conclusive agreement about the best treatment solution in terms of functional and esthetic needs of the patient was not achieved, and it involves not only orthodontists but also general dentists. Advantages and limits of treatment options are still discussed. Both treatments are long, difficult, invasive, and expensive. The perception of patients is not strictly related to standardized protocols and technical implications which certainly affect clinicians' decisions. The absence of maxillary lateral incisors could affect smile esthetics either with treatment or not. Diastemas, tipping, retention of deciduous teeth, and midline deviation could be common in these patients if not treated. In some cases, patients could prefer a less invasive treatment of tooth remodeling rather than orthodontic treatment which may be the treatment option proposed by dentists and orthodontists. Some studies [12] show that, compared to dentists and orthodontists, patients could accept a wider range of smile deviations such as midline deviation or smile with deviation from the long axes of the lateral incisors. Also, the size of smile deviation should be evaluated. Midline deviations seem to be more tolerated if less than 2 mm [13]. Alterations due to the absence of maxillary lateral incisors involve aspects of cosmetic dentistry such as tooth proportionality, contacts, connectors, and embrasures, and gingival characteristics [14], which should be taken into account for diagnosis and treatment procedures. The agenesis could be monolateral or bilateral, producing asymmetric or symmetric alterations with different treatment needs [15]. Esthetics plays an important role in managing these clinical situations, and in some cases, dentists and orthodontists emphasize its importance more than functional aspects [16].

The aim of the present study was to investigate perceptions on altered smiles due to missing maxillary lateral incisors, with or without treatment, stating the null hypothesis that no differences in terms of esthetic perception exist among different groups of people (laypersons, adult orthodontic patients, general dentists, and orthodontists).

## Methods

**Figure 1:**
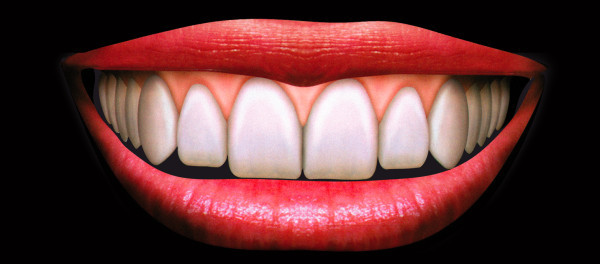
**Ideal smile.**

An ideal smile model was selected [17] (Figure 1) with the purpose of making a template digitally manipulated in all its components. The selected picture was edited using Adobe Photoshop CS3 software (Adobe Systems, San Jose, CA, USA). Graphic components were carried out from the picture of the ideal smile: upper lip, lower lip, gingival tissue, and teeth. Each component was editable in position, size, and shape in order to simulate different clinical situations and treatment options.

**Figure 2:**
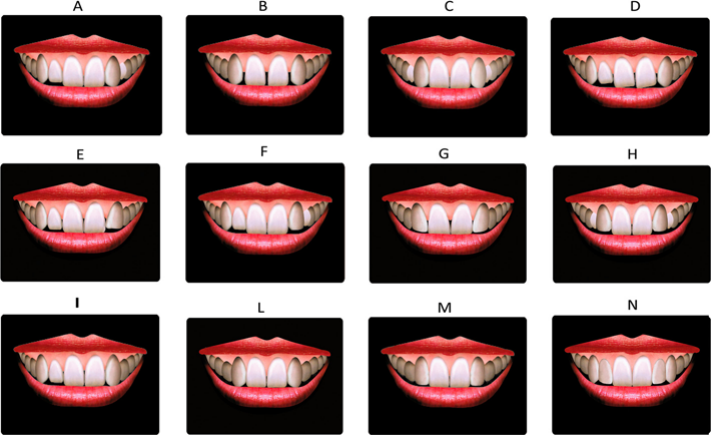
**Simulations of different smiles and treatment options.** Descriptions are found in the text.

Twelve treatment option simulations (Figure 2) with monolateral or bilateral missing lateral incisors were used in the study as follows:

- Simulation A is monolateral agenesis (2.2) with the permanent canine at the side of the agenesis and the deciduous canine (6.3) in the place of the permanent. The harmony of the smile was maintained, as well as the size of the connectors, and no diastemas were noticeable.
- Simulation B is bilateral agenesis (1.2 and 2.2) without deciduous canines (5.3 and 6.3) and diastemas between the front teeth.
- Simulation C is bilateral agenesis with persistence of the deciduous canines in the place of the permanent.
- Simulation D is monolateral agenesis (2.2) without deciduous canine (6.3). Diastemas, dental midline deviation, and tipping of the teeth are evident in this simulation due to teeth migration in the area of the missing lateral incisor.
- Simulation E is monolateral agenesis (2.2) without deciduous canine (6.3), with restoration of the front teeth and grinding of the canine (2.3) in order to eliminate spaces and diastemas. The midline is deviated to the agenesis side, and the restorations are oversized in order to fill diastemas.
- Simulation F is monolateral agenesis (2.2) with deciduous canine (6.3), with restoration of the frontal teeth and grinding of the canine (2.3) in order to eliminate spaces or diastemas. Due to the persistence of the deciduous canine, the midline is correct and the restoration is not oversized.
- Simulation G is bilateral agenesis (1.2 and 2.2) without deciduous canines, with simulation of anterior teeth esthetic restorations. These restorations are intended to correct diastemas, but they are oversized and do not fit correct smile proportions regarding crown size and gingival tissues.
- Simulation H is bilateral agenesis (1.2 and 2.2) with persistence of the deciduous canines and restorations of the front teeth and grinding of the cusps of the permanent canines.
- Simulation I is monolateral agenesis (2.2) and space closure treatment. The midline was shifted with no tipping, and gingival margins are maintained.
- Simulation L is bilateral agenesis (1.2 and 2.2) and space closure treatment. The midline is correct.
- Simulation M is bilateral agenesis (1.2 and 2.2) with space closure treatment and grinding of the canine cusps.
- Simulation N is bilateral agenesis (1.2 and 2.2) with space closure treatment and grinding of the canine cusps, with bleaching of the canine crowns and correction of the gingival levels.

All the simulations were collated on the second page of a two-page survey. Prints of the pictures were 2.4 × 3.1 in. in size and were produced with the same inkjet printer on photo-quality glossy paper, using the 1,400-dpi print mode. Subjects (160) belonging to the four categories of people were selected for the interview: 40 laypeople (N), 40 adult orthodontic patients (P), 40 general dentists (D), and 40 orthodontists (S). Orthodontic patients were recruited at the dental clinic of the University of Insubria (Varese, Italy), while general dentists and orthodontists were recruited among former/current undergraduate/postgraduate students and associates of the dental school. Finally, laypeople were recruited among those accompanying relatives to the dental clinic who were not undergoing orthodontic treatments. The age of the whole sample ranged between 25 and 60 years old and showed a similar socioeconomic status. Ethical approval for this study was obtained from the local ethical committee of the University of Insubria (no. 5184), and informed consent was obtained from the involved subjects.

The first page of the questionnaire was different depending on the category of the responder, collecting demographic data (age, sex, and income) for laypeople and also educational information for dentists and orthodontists. A visual analog scale (VAS) of 100 mm was drawn below each graphic simulation. Each respondent was asked to mark with a cross the value corresponding to each simulation. The distance from the most external left point of the line to the marked cross was measured with an Ultra-Cal Mark III (Fred V. Fowler, Newton, MA, USA) digital caliper by the same trained operator. The scale was divided into units ranging from 0 to 100. Numerical scale allowed easier collection and analysis of data. Moreover, each respondent was asked to rank in ascending order all the simulations presented, starting from the least attractive in his perception. This index allowed for descriptive analysis.

### Sample size calculation

A sample size of at least 35 subjects from each group was set to detect an effect size coefficient [18] for the VAS score of 0.8 among them, with an alpha set at 0.05 and a power of 0.8 [19]. An effect size of at least 0.8 is regarded as a 'large effect’ [18].

### Statistical analysis

The Statistical Package for Social Sciences Software release 13.0 (SPSS Inc., Chicago, IL, USA) was used for data analysis. After testing the normality of the data with the Shapiro-Wilk test and Q-Q normality plots and the equality of variance among the datasets using a Levene test, parametric methods were used for data analysis.

A one-way analysis of variance (ANOVA) was used to assess the significance of the differences in VAS scores among the groups for each simulation. When significant interactions were seen, an independent sample *t* test was employed for pairwise comparisons among all the possible combinations of the groups. In particular, each of the retrieved *p* values was multiplied for six. A *p* value less than 0.05 was used in the rejection of the null hypothesis.

## Results

The VAS scores for each group, according to simulation, are summarized in Table 1. Significant differences resulting from the ANOVAs among the groups were seen for simulations B, D, H, I, L, and N. No significant differences were seen for all of the other simulations. Moreover, pairwise analyses showed significant differences for categories D, N, and P when compared to category S (simulations B and L), for categories N and P when compared to category S (simulation H and simulation I) and to category D (simulation I), and for category P when compared to category S (simulations D and N) (Table 1).

**Table 1:** **The VAS scores (as mean ± SD) for each group, according to simulation**

Category	Sim A	Sim B	Sim C	Sim D	Sim E	Sim F	Sim G	Sim H	Sim I	Sim L	Sim M	Sim N
Dentists (D)	28.0 ± 19.9	19.3^a^ ± 15.7	46.9 ± 18.9	53.9 ± 18.2	55.1 ± 14.1	36.1 ± 21.2	59.8 ± 21.4	73.2 ± 16.5	93.4 ± 6.59	8.3^a^ ± 9.1	31.4 ± 13.4	33.7 ± 17
Laypeople (N)	34.3 ± 22.4	20.7^a^ ± 19.9	50.4 ± 21	46.1 ± 21.8	50.8 ± 18.2	44.1 ± 22	66.3 ± 18.9	75.3^a^ ± 18.8	84.2^a,b^ ± 17.2	7.4^a^ ± 7.9	32.5 ± 13.4	33.7 ± 19.2
Patients (P)	36.4 ± 22.6	21.3^a^ ± 19.7	46.3 ± 24.8	41^a^ ± 22.3	52.6 ± 22.4	43 ± 20.7	60.5 ± 24.3	77.5^a^ ± 21.6	85.2^a,b^ ± 17.9	10.6^a^ ± 10.3	30.8 ± 20.9	32.1^a^ ± 18.3
Orthodontists (S)	29.9 ± 13.3	28.8 ± 16.9	47.5 ± 17.7	56.8 ± 18.5	54.4 ± 16.9	41.3 ± 15.4	58.4 ± 19.1	69.6 ± 19.2	93.1 ± 8.9	20.3 ± 13.6	33.2 ± 14	42.1 ± 15.3

*N* = 40 for each group. Significant differences (*p* < 0.05) resulting from ANOVA and post hoc with pairwise comparison analysis are displayed. ^a^Significant compared to category S; ^b^significant compared to category D.

**Figure 3:**
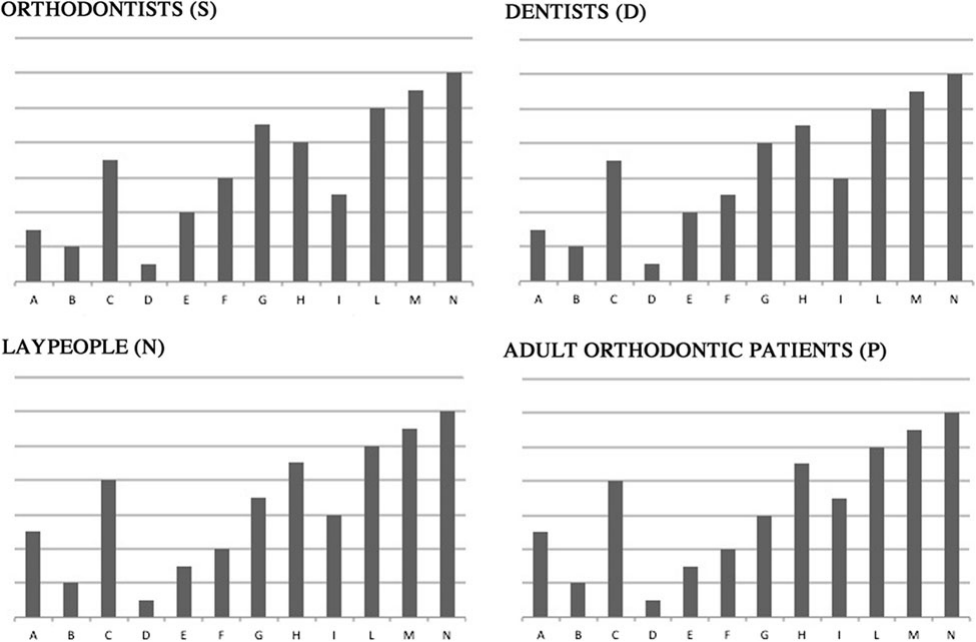
**Descriptive analyses.** Rate of appreciation of simulations for each category of evaluators (S, D, N, and P) when they were asked to rank simulations in ascending order from the least to the most pleasant.

Descriptive analyses showing the ranking of appreciation of simulations in ascending order across each group are shown in Figure 3. Among all simulations and groups, the assessment of extreme ratings, both positive and negative, was fully consistent. In particular, simulations D and B were ranked as the least attractive, while simulations L, M, and N were ranked as the most attractive.

## Discussion

Among all simulations and groups, the assessment of extreme ratings, both positive and negative, was fully consistent. Other studies found significant differences in smile perceptions when different categories of people were asked to judge the esthetics of smile [20].

All categories of the sample ranked simulations D and B as the most unpleasant. The presence of dental tipping and marked diastema in the arch, even in the presence of symmetrical smile (simulation B), were disharmonious aspects less tolerated in a smile by all categories of evaluators, although orthodontists seem to tolerate these factors much more than the other categories. Other studies showed how diastemas could compromise esthetics of smile [15, 21]. This aspect is strictly connected to the sense of unity of a smile which could play an important role even when compared with other esthetic principles such as harmony and balance [12, 22, 23].

Symmetrical smiles (simulations C, G, and H) were considered more attractive than asymmetrical simulations (simulations A, E, F, and I) by all respondents. Asymmetric alterations could make teeth more unattractive to dental professionals and laypersons, as shown by other authors [15].

All categories also tolerated persistence of deciduous teeth in the arch even more in the case of symmetric solutions (simulation A was ranked lower than simulation C by all the categories of respondents) and if placed next to the canines with its cusp camouflaged (simulation H more accepted than simulation C in all four groups). All simulations associated with orthodontic treatment (L, M, and N) were at the top of the rank, considering attractiveness of smile for all the four categories, except for simulation I, probably due to the asymmetry and midline shift. Simulation L indicated space closure treatment in the case of bilateral agenesis, and it was ranked lower than simulations M and N where other variables were modified such as restorative and periodontal procedures. This is maybe due to the fact that, even in the drawings, anatomical differences between the lateral incisors and canines are evident also for patients and laypeople. The lateral incisor is smaller and flat-faced, while the canine is more conical. Hence, when closing space option is chosen, clinicians should plan cosmetic procedures at the end of orthodontic treatment to make the canines look more similar to the lateral incisors' shape. The option considered most appreciable was simulation N. In this picture, space closure with camouflage and bleaching of relocated canines and periodontal and gingival margins correction was simulated. Space closure treatment with grinding of cuspids (simulation M) was significantly considered as more attractive by laypeople and adult orthodontic patients when compared to orthodontists. Clinicians often suggest this treatment option, but they could tend to prefer more ideal options (simulation N) considering factors which may not affect judgment of laypeople and patients.

Simulation N was ranked higher than simulations M and L maybe because of both the bleaching and periodontal correction. In fact, other studies underlined the importance of teeth color in the perception of smile [24–26]. Even though drawings were used in this study, difference in color was appreciable. Furthermore, gingival and periodontal contouring together with restorations showed more balance and harmony in a smile, leading to the ideal proportions and shapes [14, 27]. Orthodontists and dentists assigned values significantly higher than patients and laypeople for simulation N, indicating that dental professionals could emphasize the ideal treatment solutions more than patients really understand these choices.

Clinical pictures were not used in this study but drawings of different smile simulations. They could be considered less natural and less beautiful than simulations performed on clinical photos but allow better overcoming of some variables of a picture. Furthermore, patient anatomical and morphological traits which could influence esthetic judgment were eliminated. Hence, drawings were chosen for more reliability and usefulness in the aim of this study that was not to define the absolute pleasantness of a smile but rather to compare different situations of the same smile combining its components. Nevertheless, this choice could be considered a limitation of our study.

Sex and age have been considered to influence people's perceptions of smiles [28–30], but some studies did not confirm these results [12]. The present study lacks this additional evaluation that will be hopefully taken into account in further studies. Moreover, in this study, adult orthodontic patients were considered as a separate group from other laypeople. Indeed they could be closer to orthodontics than laypeople who have not undergone orthodontic treatment because of their appointments and their meetings with the clinician who explains to them treatment planning or progresses. On account of this, their smile perception could be affected by other orthodontic knowledge acquired during treatment. Our results should be interpreted as an average assessment of different groups of raters, but not as a major indicator in choosing a treatment option over another, without having evaluated all clinical and esthetic advantages and disadvantages in every single patient.

## Conclusions

The null hypothesis is rejected.

- Significant differences in smile perceptions were found between professionals (dentists and orthodontists), orthodontic patients, and laypeople, indicating that different views on smile esthetics could take place in different categories of people, maybe due to professionals' tendency toward ideal situations.
- Orthodontic treatment, absence of diastema, and symmetry were found to be characteristics of smile simulations ranked highest by all four categories.

## Abbreviations

ANOVA: One-way analysis of variance
VAS: Visual analog scale.
